# Pelvic organ prolapse in nulliparae

**DOI:** 10.1111/ajo.13481

**Published:** 2022-01-20

**Authors:** Hans Peter Dietz, Leilani Chavez‐Coloma, Talia Friedman, Friyan Turel

**Affiliations:** ^1^ University of Sydney Sydney NSW Australia; ^2^ Department Of Obstetrics and Gynecology Dr. Jose R. Reyes Memorial Medical Center Manila Philippines; ^3^ Sheba Medical Center Tel Aviv University Tel Aviv Israel

**Keywords:** childbirth, cystocele, pelvic floor disorders, pelvic organ prolapse, rectocele, uterine prolapse

## Abstract

**Background:**

Pregnancy and childbirth are thought to be the strongest environmental risk factors for pelvic organ prolapse, but prolapse does occur in nulliparae.

**Aim:**

To characterise prolapse in vaginal nulliparae.

**Material and methods:**

This was a retrospective study using archived clinical and imaging data of 368 vaginally nulliparous women seen between 2006 and 2017 at two tertiary urogynaecological centres. Patients underwent a standardised interview, clinical examination and 3D/4D translabial ultrasound. Volume datasets were analysed by the second author, blinded against all clinical data, using post‐processing software on a personal computer. Significant prolapse was defined as Pelvic Organ Prolapse Quantification system stage ≥2 for the anterior and posterior compartment, and stage ≥1 for the central compartment. On imaging, significant prolapse was defined as previously described.

**Results:**

Of 4297 women seen during the inclusion period, 409 were vaginally nulliparous, for whom 368 volume data sets could be retrieved. Mean age was 50 years (17–89) and mean body mass index 29 (16–64). Eighty‐one (22%) presented with prolapse symptoms. On clinical examination, 106 women (29%) had significant prolapse, mostly of the posterior compartment (*n* = 70, 19%). On imaging 64 women showed evidence of significant prolapse (17%), again mostly posterior (*n* = 47, 13%). Rectovaginal septal defects were even more common in 69 (19%). On multivariate analysis we found no differences between true nulliparae (*n* = 184) and women delivered exclusively by caesarean section (*n* = 184).

**Conclusions:**

Prolapse occurs in vaginal nulliparae, but it has distinct characteristics. Rectocele predominates, while cystocele and uterine prolapse are uncommon. Pregnancy and caesarean delivery seem to have little effect.

## INTRODUCTION

Pelvic organ prolapse is associated with parity, mode of delivery, age and body mass index (BMI).^1^, ^2^, ^3^ Pregnancy and childbirth are thought to be the strongest environmental risk factors for prolapse.^4^ However, prolapse does occur in nulliparae,^5^, ^6^ although it is much less common. In the 1950s and ’60s it was regarded as unusual enough to prompt case reports in the medical literature.^7^ To our knowledge, there have been very few studies on prolapse in nulliparae,^8^ and even fewer have used imaging to obtain measures of organ support. Usually, such studies involve prolapse questionnaires^9^, ^10^, ^11^ and evaluation of surface anatomy by clinical examination.^12^

It has previously been shown that defects of the rectovaginal septum, that is, diverticula of the rectal ampulla developing into the vagina, are not that uncommon even in young, nulligravid^13^ and nulliparous pregnant women^14^ as well as in older nulliparous women suffering from symptoms of pelvic floor dysfunction,^15^ and rectocele in nulliparae has been recognised for at least two decades.^16^ Imaging studies using ultrasound and magnetic resonance imaging have shown there is great variation both in organ descent and hiatal distensibility,^17^, ^18^, ^19^ and that there also seems to be inter‐ethnic variation in organ support in nulliparae.^20^

In this observational study, we attempted to determine the prevalence of signs and symptoms of prolapse among vaginally nulliparous women presenting to a urogynaecology unit and to describe associated symptoms and findings on prolapse assessment by clinical examination and translabial ultrasound.

## MATERIALS AND METHODS

This is a retrospective analysis of 368 vaginally nulliparous women seen routinely at two tertiary urogynaecological centres between November 2006 and June 2017. Women after failed attempts at vaginal operative delivery were excluded. All patients underwent a physician‐directed standardised interview, clinical examination and 3D/4D translabial ultrasound.^21^ Prolapse symptoms were defined as the sensation (feeling or seeing) of a lump or bulge in the vagina, or a dragging sensation. Such symptoms were quantified by visual analogue scale (VAS) from zero to ten as previously validated by us.^22^ Obstructed defecation symptoms (incomplete bowel emptying, straining at stool and vaginal, perineal or anal digitation) were obtained in a subset of 249 women. On clinical examination using the Pelvic Organ Prolapse Quantification (POP‐Q) system,^23^ significant prolapse was defined as POP‐Q stage 2 for the anterior and posterior compartment, and stage 1 for the central compartment.^24^

**Figure 1:**
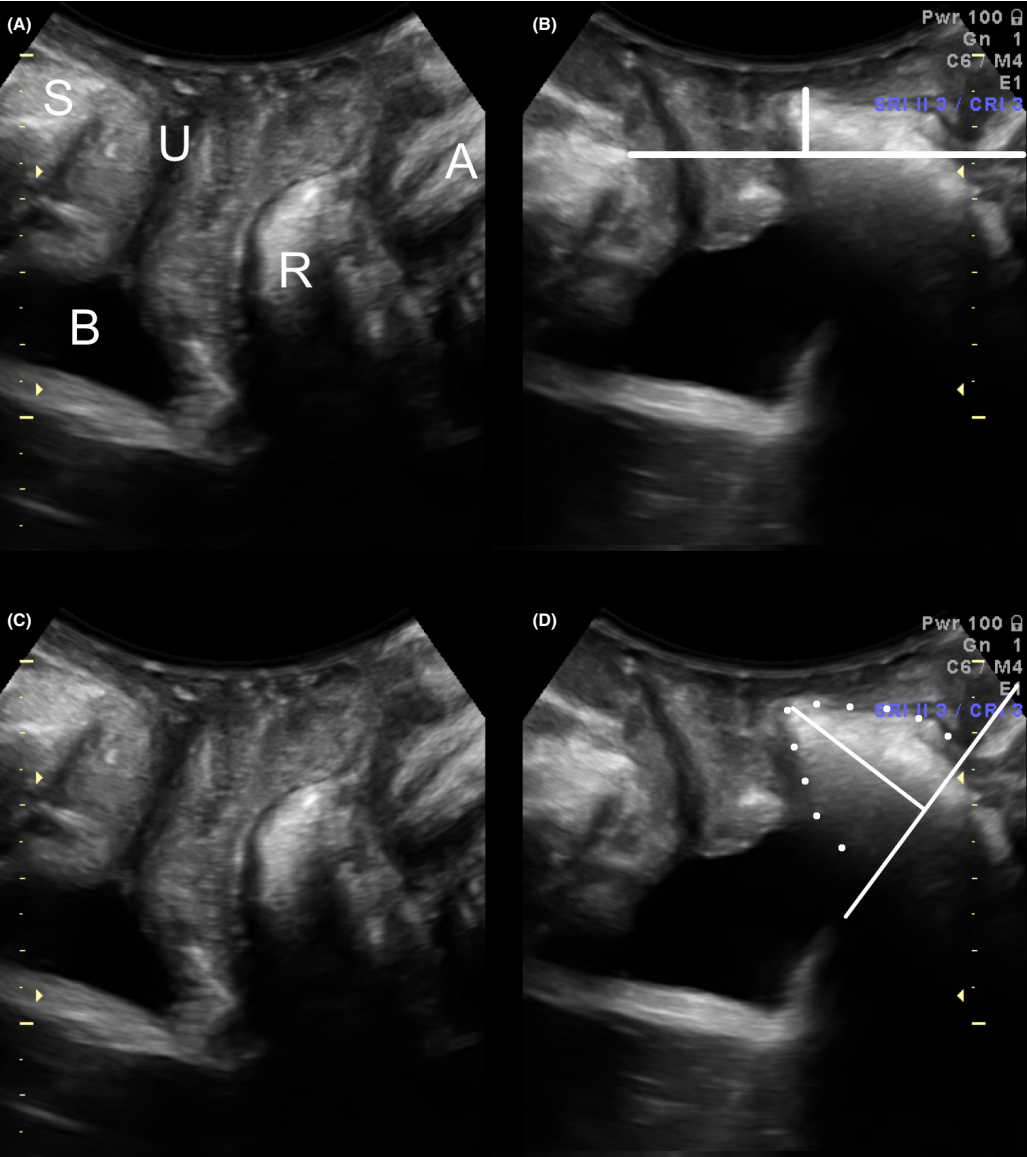
Prolapse assessment by translabial ultrasound, midsagittal plane. This is a typical small rectocele in a nulliparous patient. Panels A and C are at rest, panels B and D on Valsalva manoeuvre. The vertical line in Panel B shows descent of a small rectocele to about 1 cm below the symphyseal reference line. The oblique lines in Panel D show the base and depth of the rectocele which is outlined in dots. S, symphysis pubis; U, urethra; B, bladder; R, rectal ampulla; A, anal canal.

**Figure 2:**
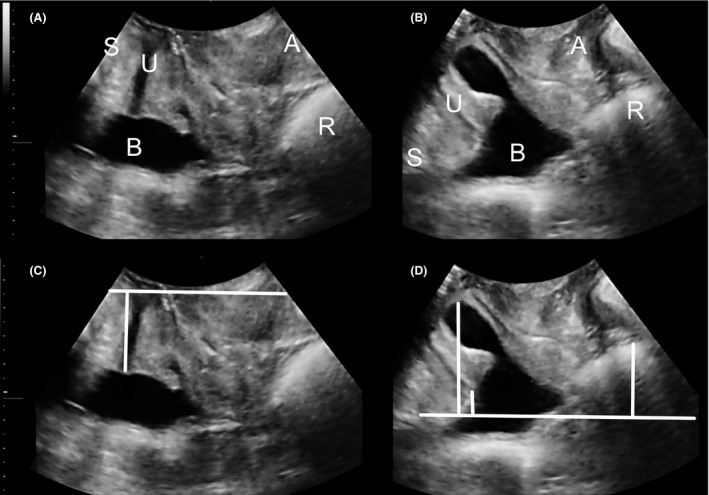
Nullipara with substantial cystocele and intact retrovesical angle. This is a much rarer situation. Panels A and C are at rest, panels B and D on Valsalva manoeuvre. The vertical line in Panel C shows the position of the bladder neck at rest, vertical lines in Panel D demonstrate (from left to right) descent of the bladder, the bladder neck and the rectal ampulla. S, symphysis pubis; U, urethra; B, bladder; R, rectal ampulla; A, anal canal.

Imaging is routinely performed with all assessments in our clinic. ‘Significant’ prolapse on imaging was defined as bladder descent to 10 mm or more below the symphysis pubis (SP), uterine descent to 15 mm or less above the SP, and rectal descent to 15 mm or more below the SP. A true rectocele (ie a defect of the rectovaginal septum) was diagnosed if there was a discontinuity of the anterior rectal muscularis layer with the internal anal sphincter on Valsalva manoeuvre, of at least 10 mm in depth.^24^ Figure 1 illustrates the commonest form of prolapse in vaginally nulliparous women, ie a ‘true’ rectocele. Figure 2 shows a much less common situation: a nullipara with a moderate cystocele and intact retrovesical angle.

Volume datasets were retrieved from storage on a server and analysed by the second author, blinded against all clinical data. Pelvic organ descent measurements and hiatal dimensions on maximum Valsalva manoeuvre were obtained offline, using post‐processing software on a personal computer. The volume data set showing the most effective Valsalva was used for analysis, ie the Valsalva manoeuvre that resulted in the greatest degree of pelvic organ descent as measured in the midsagittal plane.

This study was approved by the Nepean Blue Mountains Local Health District Human Research Ethics Committee (NBMLHD HREC 13–70). Statistical analysis was performed using SPSS v21. Chi‐squared tests were performed for statistical analysis and *P* < 0.05 was considered statistically significant. Multivariate analysis was used to control for confounders such as age, BMI, chronic constipation and obstructed defecation.

## RESULTS

Of 4297 women seen in the unit during the inclusion period, 409 were vaginally nulliparous. Ultrasound volume datasets were analysed in 368 cases as 41 datasets had missing volumes on Valsalva manoeuvre. All further analysis pertains to those 368 women of whom 184 had given birth exclusively by caesarean section, and 184 were nulliparous.

Mean age was 50 years (range 17–89), and mean BMI 29 kg/m^2^ (range 16–64). Fifteen patients (4%) had a previous incontinence or prolapse operation, 72 (20%) a hysterectomy of unknown indication. There were 175 (48%) who were menopausal. Table 1 shows demographic data for caesarean only and true nulliparae. The latter were younger on average, had a lower BMI and were less likely to have undergone a hysterectomy. Women in the caesarean only group had a median of two caesarean births (range, 1–5).

**Table 1:** Demographic data for the entire population and two subgroups (caesarean births only and true nulliparae)

	Population	Caesarean deliveries only	True nulliparae	*P*‐value
	*N* = 368	*n* = 184	*n* = 184	
Age, mean (range), years	50 (17–89)	53 (22–82)	47 (17–89)	<0.001
Body mass index, mean (range)	29 (16–64)	30 (16–49)	28 (16–64)	0.03
Previous incontinence or prolapse procedure, *n* (%)	15 (4%)	10 (5%)	5 (3%)	NS
Previous hysterectomy, *n* (%)	72 (20%)	45 (24%)	27 (15%)	0.025
Menopausal, *n* (%)	175 (48%)	95 (52%)	80 (43%)	NS

Primary complaints were stress and/or urge urinary incontinence (*n* = 239 and 237 respectively). Seventy‐five had a hysterectomy. Eighty‐one (22%) presented with symptoms of prolapse (vaginal lump and/or bulge) at a mean bother of 5.5 (range 0–10) on a VAS of 0–10. Information on obstructed defecation was recorded in 249 women, of which 138 (55%) suffered from obstructed defecation symptoms. On clinical examination, 106 women (29%) had clinically significant prolapse as defined above. Posterior compartment prolapse was most common (*n* = 70, 19%) compared to the central (*n* = 26, 9%) and anterior (*n* = 63, 17%); see Table 2.

**Table 2:** Signs and symptoms of prolapse among 368 vaginally nulliparous women, and comparison of CS only vs nulliparae

	Population *N* = 368	CS only *n* = 184	No births *n* = 184	*P*‐value, OR (CI)	Adjusted *P*‐value, OR (CI)
Symptoms of prolapse (%) (*n* = 368)	81 (22%)	47 (26%)	34 (18%)	0.1	0.2
				1.5 (0.9‐2.5)	1.51 (0.8‐2.89)
Significant prolapse on POP‐Q (*n* = 362)	106 (29%)	59/180 (33%)	47/182 (26%)	0.13	0.5
				1.4 (0.9‐2.21)	1.25 (0.70‐2.21)
Anterior compartment stage ≥2 (*n* = 363)	63 (17%)	38/181 (21%)	25/182 (14%)	0.07	0.08
				1.67 (0.96‐2.90)	1.81 (0.93‐3.55)
Uterine prolapse stage ≥1 (*n* = 293)	26 (9%)	11/139 (8%)	15/154 (10%)	0.7	0.6
				0.8 (0.35‐1.8)	0.77 (0.25‐2.35)
Posterior compartment stage ≥2 (*n* = 363)	70 (19%)	43/181 (24%)	27/182 (15%)	0.03	0.5
				1.79 (1.05‐3.05)	1.27 (0.64‐2.57)
Significant prolapse on US (*n* = 367)	71 (19%)	39/183 (21%)	32/184 (17%)	0.35	1
				1.29 (0.77‐2.16)	0.99 (0.51‐1.92)
Significant cystocele on US (*n* = 367)	14 (4%)	9/183 (5%)	5/184 (3%)	0.29	0.7
				1.85(0.61‐5.64)	1.27 (0.38‐4.29)
Significant central compartment prolapse on US (*n* = 367)	23 (6%)	11/183 (6%)	12/184 (7%)	0.84	0.94
				0.92 (0.39‐2.13)	0.96 (0.33‐2.78)
Significant rectal descent on US (*n* = 367)	46 (13%)	27/183 (15%)	19/184 (10%)	0.2	0.7
				1.50 (0.80‐2.81)	1.17 (0.52‐2.65)
True rectocele (*n* = 367)	68 (19%)	41/183 (22%)	27/184 (15%)	0.06	0.07
				1.68 (0.98‐2.87)	1.94 (0.96‐3.92)

Univariate and multivariate logistic regression controlling for age, body mass index, chronic constipation and obstructed defecation.

CI, confidence interval; CS, caesarean section; OR, odds ratio; POP‐Q, Pelvic Organ Prolapse Quantification; US, ultrasound

On imaging analysis blinded against all other data, 64 women showed evidence of significant prolapse on ultrasound (17%), and again it was mostly the posterior compartment that was affected (*n* = 47, 13%) compared to the central compartment (*n* = 12, 3%), and cystocele (*n* = 14, 4%); see Table 1. True rectocele (see Fig. 1), was even more common in 69 (19%), although many of those rectoceles remained relatively high, ie did not reach 15 mm or more below the SP, and some did not cause symptoms of prolapse. Symptoms of obstructed defecation were associated with posterior compartment descent on clinical examination (*P* = 0.001), but not with rectal descent on ultrasound.

When comparing women after exclusive caesarean section childbirth with nulliparae, symptoms of prolapse were non‐significantly more likely to be found in the caesarean section group (26% vs 18%, *P* = 0.1), and there were weak trends or marginally significant findings toward more prolapse on POP‐Q as well as more true rectocele on imaging. However, on controlling for multiple confounders (age, BMI, constipation and obstructed defecation) in multivariate analysis, these relationships all became non‐significant.

## DISCUSSION

### Main findings

This study has confirmed that pelvic organ prolapse is not rare in nulliparae seen at a urogynaecological clinic. However, prolapse in women who have not given birth vaginally shows some distinct characteristics. Cystocele seems to be uncommon in nulliparae, but this is not the case for posterior compartment descent and true rectocele, an observation that is consistent with reports on rectocele in young nonpregnant nulliparous women^10^ and in pregnant nulliparae in late gestation.^15^

We found no substantive differences in symptoms, clinical or imaging signs of prolapse when comparing women after exclusive caesarean section childbirth with nulliparae. While our results suggest a weak effect of pregnancy, such could not be conclusively demonstrated in this study. This implies that any consistent long‐term effect of pregnancy itself or its hormonal milieu on symptoms or signs of prolapse is bound to be small and unlikely to be clinically significant. Giving birth exclusively by caesarean section largely seems to preserve a nulliparous pelvic floor and pelvic organ support.

### Strengths and limitations

The greatest strength of this study is the large population size, which allowed investigation of prolapse symptoms and signs in 368 women who had never given birth vaginally. Another strength is the imaging methodology which has been internationally standardised,^25^ and the blinding that is easily achieved when using imaging data sets that are analysed offline, without access to clinical data.

However, there are several weaknesses to this study that should be acknowledged. Firstly, this was a retrospective study without power calculations since pilot data to inform such calculations could not be sourced. In addition, we utilised data obtained in routine clinical urogynaecological practice. This implies that our population suffers from substantial selection bias, likely constituting an enriched sample. Further studies in nulliparous women should be performed on unselected cohorts. In addition, the patients analysed for this study were relatively young when compared to women typically presenting for prolapse. Also, some may question the inclusion of women exclusively delivered by caesarean, but we present data for both true nulliparae and those delivered only by caesarean and have not been able to show any significant differences between these two groups. On comparing true nulliparae and those delivered by caesarean, analysis is limited by the obvious fact that the two groups differ in important demographic descriptors such as age, BMI and previous hysterectomy. While we tried to account for such confounders by using multivariate analysis, such comparisons need to be interpreted with caution. Finally, our patients were largely Caucasian, limiting the applicability of these findings in other ethnic groups. This is particularly relevant in view of the emerging data on inter‐ethnic variability of functional pelvic floor anatomy,^26^, ^27^, ^28^ suggesting that studies in other ethnic groups would be useful to further investigate this research question.

### Interpretation

A comparison of our data with the literature suffers from the fact that much work on prolapse has used questionnaires as the main assessment tool, implying that a distinction between the different compartments is impossible.^10^, ^11^ There are very few studies providing clinical prolapse assessment data in nulliparae, and they tend to be very small^9^, ^12^ and/or provide global staging only, rather than compartment‐specific data.^5^ Over the last decade a number of imaging studies in nulliparous women have been published, utilising both magnetic resonance^18^ and ultrasound imaging techniques,^8^, ^17^, ^18^, ^19^, ^20^, ^26^ but they focus on the levator ani and/or report on series that are too small to inform on prolapse, a condition that is plainly of low prevalence in nulliparae. We are not aware of any other study in the literature that focuses on prolapse in nulliparous women.

In conclusion, pelvic organ prolapse is not a rare finding in vaginally nulliparous women presenting for urogynaecological assessment. Prolapse in vaginal nulliparae, whether truly nulliparous or only delivered by caesarean section, is most likely to be found in the posterior compartment. Uterine prolapse and cystocele are comparatively rare.

## AUTHOR CONTRIBUTIONS

Hans Peter Dietz: project conception, design and development; data acquisition; analysis and interpretation; drafting the manuscript, revising it critically for important intellectual content and final approval of the version to be published. Leilani Chavez Coloma: data acquisition; analysis and interpretation; drafting the manuscript, revising it critically for important intellectual content and final approval of the version to be published. Talia Friedman: data acquisition; analysis and interpretation; drafting the manuscript, revising it critically for important intellectual content and final approval of the version to be published. Friyan Turel: data acquisition; analysis and interpretation; drafting the manuscript, revising it critically for important intellectual content and final approval of the version to be published.

## Funding

Open access publishing facilitated by The University of Sydney, as part of the Wiley ‐ The University of Sydney agreement via the Council of Australian University Librarians.
